# 11,12-Dihy­droxy-10,6,8,11,13-icetexapentan-1-one

**DOI:** 10.1107/S1600536810053754

**Published:** 2011-01-08

**Authors:** Ibrahim Abdul Razak, Suchada Chantrapromma, Abdul Wahab Salae, Hoong-Kun Fun

**Affiliations:** a X-ray Crystallography Unit, School of Physics, Universiti Sains Malaysia, 11800 USM, Penang, Malaysia; b Crystal Materials Research Unit, Department of Chemistry, Faculty of Science, Prince of Songkla University, Hat-Yai, Songkhla 90112, Thailand

## Abstract

The title compound [systematic name: 14,15-dihy­droxy-7,7-dimethyl-13-(propan-2-yl)tricyclo­[9.4.0.0^3,8^]penta­deca-1(11),3(8),9,12,14-pentaen-4-one], C_20_H_24_O_3_, is a new icetexane diterpenoid which was isolated from the roots of *Premna obtusifolia* (Verbenaceae). The mol­ecule has three fused rings: a cyclo­hexenone, a central cyclo­heptene and a benzene ring. The cyclo­hexenone ring is in an envelope conformation, whereas the cyclo­heptene ring is in a twisted boat conformation. Intra­molecular O—H⋯O hydrogen bonds generate *S*(5) and *S*(8) ring motifs. In the crystal, mol­ecules are linked into dimers through O—H⋯O hydrogen bonds. These dimers are arranged in to sheets parallel to the *ac* plane. C—H⋯O and weak C—H⋯π inter­actions are also present.

## Related literature

For details of hydrogen-bond motifs, see: Bernstein *et al.* (1995 ▶) and for ring conformations, see: Cremer & Pople (1975 ▶). For bond-length data, see: Allen *et al.* (1987 ▶). For background to Verbenaceae plants and the bioactivity of icetexane, see: Bunluepuech & Tewtrakul (2009 ▶); Hymavathi *et al.* (2009 ▶); Simmons & Sarpong (2009 ▶). For related structures, see: Asik *et al.* (2010 ▶); Razak *et al.* (2010 ▶). For the stability of the temperature controller used in the data collection, see Cosier & Glazer (1986 ▶).
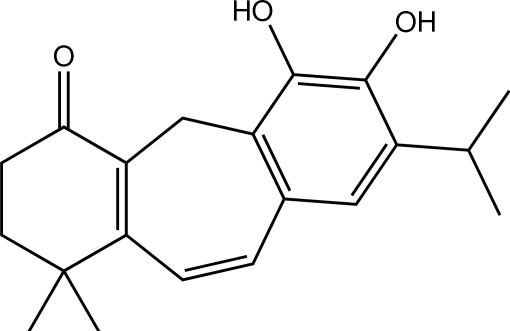


## Experimental

### 

#### Crystal data

C_20_H_24_O_3_
*M* *_r_* = 312.39
Monoclinic, 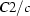

*a* = 25.1090 (9) Å
*b* = 9.4317 (3) Å
*c* = 14.9609 (4) Å
β = 108.683 (2)°
*V* = 3356.35 (19) Å^3^
*Z* = 8
Mo *K*α radiation
μ = 0.08 mm^−1^
*T* = 100 K
0.60 × 0.32 × 0.28 mm

#### Data collection

Bruker APEXII CCD area-detector diffractometer
Absorption correction: multi-scan (*SADABS*; Bruker, 2005 ▶) *T*_min_ = 0.953, *T*_max_ = 0.977
60861 measured reflections
7404 independent reflections
6198 reflections with *I* > 2σ(*I*)
*R*_int_ = 0.029

#### Refinement

*R*[*F* ^2^ > 2σ(*F* ^2^)] = 0.042
*wR*(*F* ^2^) = 0.125
*S* = 1.03
7404 reflections
304 parameters
All H-atom parameters refined
Δρ_max_ = 0.48 e Å^−3^
Δρ_min_ = −0.21 e Å^−3^

### 

Data collection: *APEX2* (Bruker, 2005 ▶); cell refinement: *SAINT* (Bruker, 2005 ▶); data reduction: *SAINT*; program(s) used to solve structure: *SHELXTL* (Sheldrick, 2008 ▶); program(s) used to refine structure: *SHELXTL*; molecular graphics: *SHELXTL*; software used to prepare material for publication: *SHELXTL* and *PLATON* (Spek, 2009 ▶).

## Supplementary Material

Crystal structure: contains datablocks global, I. DOI: 10.1107/S1600536810053754/ng5091sup1.cif

Structure factors: contains datablocks I. DOI: 10.1107/S1600536810053754/ng5091Isup2.hkl

Additional supplementary materials:  crystallographic information; 3D view; checkCIF report

## Figures and Tables

**Table 1:** Hydrogen-bond geometry (Å, °) *Cg*1 is the centroid of C8–C9/C11–C14 ring.

*D*—H⋯*A*	*D*—H	H⋯*A*	*D*⋯*A*	*D*—H⋯*A*
O2—H1*O*2⋯O1	0.870 (18)	2.088 (18)	2.9479 (8)	169.8 (15)
O2—H1*O*2⋯O2^i^	0.870 (18)	2.541 (16)	2.8818 (7)	104.3 (12)
O3—H1*O*3⋯O2	0.875 (14)	2.208 (16)	2.6955 (7)	114.9 (12)
O3—H1*O*3⋯O1^i^	0.875 (14)	2.046 (14)	2.8448 (7)	151.3 (14)
C7—H7*A*⋯O2^ii^	0.974 (12)	2.440 (12)	3.2262 (9)	137.5 (10)
C15—H15*A*⋯O3	1.007 (15)	2.364 (15)	2.8216 (8)	106.6 (10)
C18—H18*B*⋯O3^iii^	0.986 (15)	2.585 (15)	3.3467 (10)	134.1 (11)
C19—H19*B*⋯*Cg*1^ii^	1.011 (15)	2.798 (16)	3.7130 (10)	150.8 (12)
C20—H20*A*⋯*Cg*1^iv^	0.993 (11)	2.847 (12)	3.7506 (8)	151.6 (9)

Symmetry codes: (i) 
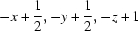
; (ii) 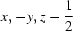; (iii) 
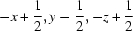
; (iv) 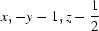.

**Fig. 1.**
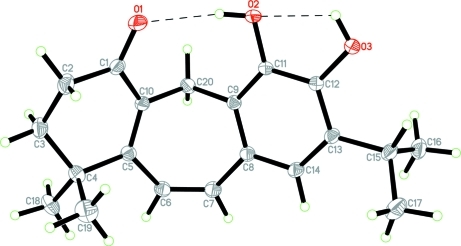
The structure of (I), showing 50% probability displacement ellipsoids and the atom-numbering scheme. Intramolecular O—H···O hydrogen bonds are shown as dashed lines.

**Fig. 2.**
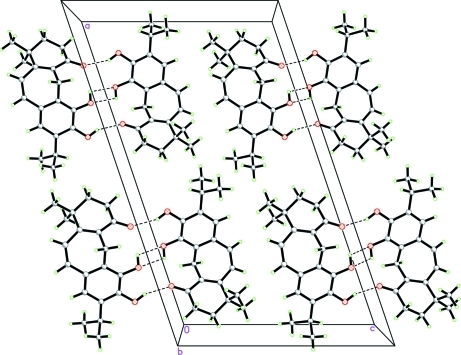
The crystal packing of (I) viewed along the b axis, showing sheets parallel to the ac plane. Hydrogen bonds are shown as dashed lines.

## supplementary crystallographic information

### Comment

The extracts of Verbenaceae plants have been found to possess anti-HIV-1 integrase activity (Bunluepuech & Tewtrakul, 2009). *Premna obtusifolia* (Verbenaceae), a small tree found in the mangrove forests, is one of the Verbenaceae plants. As part of our research on bioactive compounds from medicinal plants, we previouly reported the crystal structures of diterpenoids from the roots of *Premna obtusifolia* (Verbenaceae) which was collected from Satun province in the southern of Thailand (Asik *et al.*, 2010; Razak *et al.*, 2010). The title icetexane diterpenoid (I), also named as Obtusin N, is a new compound which was isolated from the same plant. The icetexane diterpenoids encompass a variety of bioactive and structurally interesting compounds (Hymavathi *et al.*, 2009; Simmons & Sarpong, 2009). We herein report the crystal structure of (I).

The molecule of (I) has a tricyclic skeleton (Fig. 1). The cyclohexene ring (C1–C5/C10) is in an envelope conformation with the puckering C3 atom having a deviation of 0.3373 (9) Å and puckering parameters Q = 0.4877 (9) Å, θ = 65.14 (19)° and φ = 113.04 (11)° (Cremer & Pople, 1975) whereas the central cycloheptene ring (C5–C10/C20) is in twisted-boat conformation with the most puckering atom C20 having deviation of 0.5665 (8) Å and puckering parameter Q = 0.8294 (8) Å. The benzene ring (C8–C9/C11–C14) is slightly twisted with the maximum deviation of -0.0575 (7) and 0.0388 (7) Å for atoms C9 and C11, respectively. The two hydroxy groups are co-planar with the attached benzene ring with *r.m.s.* deviation of 0.026 (7) Å. The orientation of the propanyl group is described by the torsion angles C14–C13–C15–C16 = 81.62 (9)° and C14–C13–C15–C17 = -42.19 (10)°. Intramolecular O3—H1O3···O2 and O2—H1O2···O1 hydrogen bonds (Table 1) generate S(5) and S(8) ring motifs, respectively (Fig. 1) (Bernstein *et al.*, 1995). The bond distances in (I) are within normal ranges (Allen *et al.*, 1987) and comparable to the related structures (Asik *et al.*, 2010; Razak *et al.*, 2010).

The crystal packing of (I) is stabilized by intermolecular O—H···O hydrogen bonds, C—H···O and C—H···π weak interactions (Fig. 2 and Table 1). The molecules are linked into dimers through O3—H1O3···O1 hydrogen bonds (Table 1 and Fig. 2). These dimers are arranged into sheets parallel to the *ac* plane. C—H···π weak interactions were presented (Table 1).

### Experimental

The air-dried roots of *premna obtusifolia* (4.5 kg) were extracted with hexane (2 *x* 20 *L*) at room temperature. The combined extracts were concentrated under reduced pressure to afford a dark yellow extract (40.0 g) which was subjected to quick column chromatography (QCC) over silica gel using solvents of increasing polarity from n-hexane to EtOAc to afford 7 fractions (F1—F7). Fraction F6 was further purified by quick column chromatography (QCC) using n-hexane-ETOAc (9:1), yielding the title compound (87.3 mg). Yellow needle-shaped single crystals of the title compound suitable for *x*-ray structure determination were recrystallized from n-hexane after several days.

### Refinement

All H atoms were located in a difference maps and isotropically refined. A rotating group model was used for the methyl groups. The highest residual electron density peak is located at 0.64 Å from C8 and the deepest hole is located at 1.04 Å from C10.

### Crystal data

**Table:**

C_20_H_24_O_3_	*F*(000) = 1344
*M**_r_* = 312.39	*D*_x_ = 1.236 Mg m^−^^3^
Monoclinic, *C*2/*c*	Mo *K*α radiation, λ = 0.71073 Å
Hall symbol: -C 2yc	Cell parameters from 7404 reflections
*a* = 25.1090 (9) Å	θ = 2.3–35.0°
*b* = 9.4317 (3) Å	µ = 0.08 mm^−^^1^
*c* = 14.9609 (4) Å	*T* = 100 K
β = 108.683 (2)°	Needle, yellow
*V* = 3356.35 (19) Å^3^	0.60 × 0.32 × 0.28 mm
*Z* = 8	

### Data collection

**Table:**

Bruker APEXII CCD area-detector diffractometer	7404 independent reflections
Radiation source: sealed tube	6198 reflections with *I* > 2σ(*I*)
graphite	*R*_int_ = 0.029
φ and ω scans	θ_max_ = 35.0°, θ_min_ = 2.3°
Absorption correction: multi-scan (*SADABS*; Bruker, 2005)	*h* = −38→40
*T*_min_ = 0.953, *T*_max_ = 0.977	*k* = −14→15
60861 measured reflections	*l* = −24→24

### Refinement

**Table:**

Refinement on *F*^2^	Primary atom site location: structure-invariant direct methods
Least-squares matrix: full	Secondary atom site location: difference Fourier map
*R*[*F*^2^ > 2σ(*F*^2^)] = 0.042	Hydrogen site location: inferred from neighbouring sites
*wR*(*F*^2^) = 0.125	All H-atom parameters refined
*S* = 1.03	*w* = 1/[σ^2^(*F*_o_^2^) + (0.0755*P*)^2^ + 1.0087*P*] where *P* = (*F*_o_^2^ + 2*F*_c_^2^)/3
7404 reflections	(Δ/σ)_max_ = 0.001
304 parameters	Δρ_max_ = 0.48 e Å^−^^3^
0 restraints	Δρ_min_ = −0.21 e Å^−^^3^

### Special details

**Table:**

Experimental. The crystal was placed in the cold stream of an Oxford Cryosystems Cobra open-flow nitrogen cryostat (Cosier & Glazer, 1986) operating at 100.0 (1) K.
Geometry. All esds (except the esd in the dihedral angle between two l.s. planes) are estimated using the full covariance matrix. The cell esds are taken into account individually in the estimation of esds in distances, angles and torsion angles; correlations between esds in cell parameters are only used when they are defined by crystal symmetry. An approximate (isotropic) treatment of cell esds is used for estimating esds involving l.s. planes.
Refinement. Refinement of F^2^ against ALL reflections. The weighted R-factor wR and goodness of fit S are based on F^2^, conventional R-factors R are based on F, with F set to zero for negative F^2^. The threshold expression of F^2^ > 2sigma(F^2^) is used only for calculating R-factors(gt) etc. and is not relevant to the choice of reflections for refinement. R-factors based on F^2^ are statistically about twice as large as those based on F, and R- factors based on ALL data will be even larger.

### Fractional atomic coordinates and isotropic or equivalent isotropic displacement parameters (Å^2^)

**Table:**

	*x*	*y*	*z*	*U*_iso_*/*U*_eq_	
O1	0.34135 (2)	0.18997 (7)	0.45441 (4)	0.02727 (13)	
O2	0.22121 (2)	0.14713 (6)	0.42998 (3)	0.02042 (11)	
H1O2	0.2573 (7)	0.1602 (17)	0.4448 (11)	0.048 (4)*	
O3	0.11523 (2)	0.24175 (6)	0.35038 (3)	0.01917 (10)	
H1O3	0.1377 (6)	0.2421 (15)	0.4088 (10)	0.041 (4)*	
C1	0.35660 (3)	0.18067 (8)	0.38355 (5)	0.02009 (13)	
C2	0.40308 (3)	0.27126 (10)	0.37203 (6)	0.02604 (15)	
H2A	0.4322 (6)	0.2867 (15)	0.4357 (10)	0.038 (3)*	
H2B	0.3867 (6)	0.3655 (16)	0.3534 (10)	0.040 (3)*	
C3	0.42779 (3)	0.20818 (9)	0.30042 (6)	0.02461 (14)	
H3A	0.4475 (5)	0.1170 (14)	0.3235 (9)	0.031 (3)*	
H3B	0.4575 (6)	0.2728 (14)	0.2903 (9)	0.035 (3)*	
C4	0.38338 (3)	0.17810 (8)	0.20403 (5)	0.01959 (12)	
C5	0.33387 (3)	0.09795 (7)	0.21853 (5)	0.01718 (11)	
C6	0.29158 (3)	0.04077 (8)	0.13478 (5)	0.02060 (13)	
H6A	0.3058 (5)	0.0138 (14)	0.0831 (9)	0.030 (3)*	
C7	0.23475 (3)	0.03856 (8)	0.11708 (5)	0.02164 (13)	
H7A	0.2123 (5)	0.0114 (13)	0.0533 (8)	0.027 (3)*	
C8	0.20317 (3)	0.08458 (7)	0.17837 (4)	0.01703 (11)	
C9	0.22671 (3)	0.07722 (7)	0.27714 (4)	0.01548 (11)	
C10	0.32690 (3)	0.08736 (7)	0.30526 (4)	0.01682 (11)	
C11	0.19897 (3)	0.14125 (7)	0.33345 (4)	0.01495 (11)	
C12	0.14429 (3)	0.19365 (7)	0.29375 (4)	0.01505 (11)	
C13	0.11813 (3)	0.19232 (7)	0.19542 (4)	0.01734 (11)	
C14	0.14864 (3)	0.14018 (8)	0.13931 (5)	0.01940 (12)	
H14A	0.1327 (5)	0.1435 (13)	0.0691 (8)	0.029 (3)*	
C15	0.05752 (3)	0.24151 (8)	0.15458 (5)	0.02181 (13)	
H15A	0.0487 (6)	0.3020 (16)	0.2035 (10)	0.043 (4)*	
C16	0.01786 (3)	0.11352 (11)	0.13660 (7)	0.03109 (18)	
H16A	0.0246 (6)	0.0563 (16)	0.0858 (10)	0.041 (3)*	
H16B	−0.0208 (6)	0.1452 (14)	0.1209 (10)	0.038 (3)*	
H16C	0.0246 (6)	0.0539 (16)	0.1939 (10)	0.043 (4)*	
C17	0.04657 (4)	0.32889 (10)	0.06447 (7)	0.03084 (17)	
H17A	0.0749 (7)	0.4078 (17)	0.0732 (11)	0.051 (4)*	
H17B	0.0075 (6)	0.3743 (15)	0.0475 (10)	0.040 (3)*	
H17C	0.0477 (6)	0.2691 (16)	0.0078 (10)	0.043 (4)*	
C18	0.41211 (4)	0.08869 (9)	0.14665 (6)	0.02682 (15)	
H18A	0.3865 (6)	0.0729 (15)	0.0807 (10)	0.036 (3)*	
H18B	0.4239 (6)	−0.0029 (16)	0.1787 (9)	0.039 (3)*	
H18C	0.4464 (5)	0.1393 (14)	0.1442 (9)	0.034 (3)*	
C19	0.36195 (4)	0.31667 (9)	0.15095 (7)	0.03147 (17)	
H19A	0.3940 (6)	0.3712 (17)	0.1398 (11)	0.048 (4)*	
H19B	0.3440 (6)	0.3764 (17)	0.1896 (10)	0.045 (4)*	
H19C	0.3354 (6)	0.2977 (15)	0.0893 (10)	0.037 (3)*	
C20	0.28082 (3)	−0.00267 (7)	0.31829 (5)	0.01867 (12)	
H20A	0.2799 (5)	−0.0936 (12)	0.2844 (8)	0.022 (3)*	
H20B	0.2880 (4)	−0.0209 (12)	0.3870 (8)	0.022 (2)*	

### Atomic displacement parameters (Å^2^)

**Table:**

	*U*^11^	*U*^22^	*U*^33^	*U*^12^	*U*^13^	*U*^23^
O1	0.0226 (2)	0.0428 (3)	0.0156 (2)	0.0040 (2)	0.00490 (18)	−0.0048 (2)
O2	0.0181 (2)	0.0313 (3)	0.01191 (19)	0.00406 (19)	0.00491 (16)	0.00394 (17)
O3	0.0166 (2)	0.0264 (2)	0.0148 (2)	0.00275 (17)	0.00536 (16)	−0.00128 (17)
C1	0.0154 (3)	0.0273 (3)	0.0159 (3)	0.0042 (2)	0.0026 (2)	−0.0018 (2)
C2	0.0178 (3)	0.0336 (4)	0.0254 (3)	−0.0039 (3)	0.0051 (2)	−0.0100 (3)
C3	0.0174 (3)	0.0287 (4)	0.0284 (3)	−0.0002 (2)	0.0082 (2)	−0.0037 (3)
C4	0.0206 (3)	0.0185 (3)	0.0220 (3)	0.0002 (2)	0.0100 (2)	−0.0002 (2)
C5	0.0178 (3)	0.0177 (3)	0.0167 (2)	0.0012 (2)	0.0065 (2)	−0.0009 (2)
C6	0.0210 (3)	0.0246 (3)	0.0174 (3)	0.0002 (2)	0.0078 (2)	−0.0051 (2)
C7	0.0210 (3)	0.0267 (3)	0.0172 (3)	0.0002 (2)	0.0061 (2)	−0.0069 (2)
C8	0.0169 (2)	0.0185 (3)	0.0154 (2)	−0.0007 (2)	0.0048 (2)	−0.0037 (2)
C9	0.0155 (2)	0.0155 (2)	0.0158 (2)	0.00006 (19)	0.00547 (19)	0.00067 (19)
C10	0.0154 (2)	0.0197 (3)	0.0151 (2)	0.0027 (2)	0.00442 (19)	0.0006 (2)
C11	0.0157 (2)	0.0166 (2)	0.0127 (2)	0.00038 (19)	0.00469 (18)	0.00190 (19)
C12	0.0152 (2)	0.0160 (2)	0.0140 (2)	−0.00001 (19)	0.00473 (19)	−0.00030 (18)
C13	0.0156 (2)	0.0200 (3)	0.0147 (2)	0.0005 (2)	0.00235 (19)	−0.0028 (2)
C14	0.0182 (3)	0.0241 (3)	0.0143 (2)	−0.0001 (2)	0.0030 (2)	−0.0046 (2)
C15	0.0183 (3)	0.0285 (3)	0.0157 (3)	0.0051 (2)	0.0013 (2)	−0.0036 (2)
C16	0.0164 (3)	0.0421 (5)	0.0334 (4)	−0.0012 (3)	0.0060 (3)	0.0097 (3)
C17	0.0259 (4)	0.0298 (4)	0.0305 (4)	0.0017 (3)	0.0002 (3)	0.0083 (3)
C18	0.0278 (3)	0.0259 (3)	0.0338 (4)	−0.0016 (3)	0.0198 (3)	−0.0033 (3)
C19	0.0345 (4)	0.0222 (3)	0.0384 (4)	0.0030 (3)	0.0126 (4)	0.0088 (3)
C20	0.0184 (3)	0.0186 (3)	0.0201 (3)	0.0032 (2)	0.0077 (2)	0.0038 (2)

### Geometric parameters (Å, °)

**Table:**

O1—C1	1.2403 (9)	C9—C20	1.5022 (9)
O2—C11	1.3725 (8)	C10—C20	1.4976 (9)
O2—H1O2	0.869 (16)	C11—C12	1.3998 (9)
O3—C12	1.3610 (8)	C12—C13	1.4058 (9)
O3—H1O3	0.875 (14)	C13—C14	1.3948 (9)
C1—C10	1.4638 (10)	C13—C15	1.5196 (9)
C1—C2	1.5003 (11)	C14—H14A	0.997 (12)
C2—C3	1.5208 (11)	C15—C17	1.5280 (12)
C2—H2A	1.008 (14)	C15—C16	1.5329 (12)
C2—H2B	0.982 (15)	C15—H15A	1.006 (15)
C3—C4	1.5406 (11)	C16—H16A	0.989 (14)
C3—H3A	0.996 (13)	C16—H16B	0.969 (14)
C3—H3B	1.011 (13)	C16—H16C	0.993 (15)
C4—C5	1.5286 (10)	C17—H17A	1.008 (16)
C4—C19	1.5345 (11)	C17—H17B	1.026 (14)
C4—C18	1.5379 (10)	C17—H17C	1.026 (15)
C5—C10	1.3674 (9)	C18—H18A	1.001 (13)
C5—C6	1.4615 (10)	C18—H18B	0.987 (15)
C6—C7	1.3655 (10)	C18—H18C	0.996 (13)
C6—H6A	0.984 (12)	C19—H19A	1.013 (15)
C7—C8	1.4574 (9)	C19—H19B	1.011 (15)
C7—H7A	0.975 (12)	C19—H19C	0.966 (14)
C8—C9	1.4065 (9)	C20—H20A	0.993 (11)
C8—C14	1.4068 (9)	C20—H20B	1.000 (11)
C9—C11	1.3912 (9)		
			
C11—O2—H1O2	108.3 (10)	O3—C12—C13	119.36 (6)
C12—O3—H1O3	108.7 (9)	C11—C12—C13	120.46 (6)
O1—C1—C10	120.59 (7)	C14—C13—C12	118.06 (6)
O1—C1—C2	121.49 (7)	C14—C13—C15	122.54 (6)
C10—C1—C2	117.78 (6)	C12—C13—C15	119.35 (6)
C1—C2—C3	111.47 (6)	C13—C14—C8	122.04 (6)
C1—C2—H2A	109.2 (8)	C13—C14—H14A	120.7 (7)
C3—C2—H2A	112.7 (8)	C8—C14—H14A	117.3 (7)
C1—C2—H2B	106.1 (8)	C13—C15—C17	113.17 (6)
C3—C2—H2B	112.5 (8)	C13—C15—C16	109.93 (6)
H2A—C2—H2B	104.4 (11)	C17—C15—C16	110.29 (6)
C2—C3—C4	113.28 (6)	C13—C15—H15A	107.8 (8)
C2—C3—H3A	111.3 (7)	C17—C15—H15A	108.4 (9)
C4—C3—H3A	107.2 (7)	C16—C15—H15A	107.0 (9)
C2—C3—H3B	110.9 (7)	C15—C16—H16A	107.6 (8)
C4—C3—H3B	108.5 (7)	C15—C16—H16B	110.0 (8)
H3A—C3—H3B	105.3 (10)	H16A—C16—H16B	112.7 (11)
C5—C4—C19	109.16 (6)	C15—C16—H16C	111.9 (8)
C5—C4—C18	110.87 (6)	H16A—C16—H16C	109.3 (12)
C19—C4—C18	109.12 (7)	H16B—C16—H16C	105.3 (11)
C5—C4—C3	109.63 (6)	C15—C17—H17A	111.3 (9)
C19—C4—C3	110.87 (7)	C15—C17—H17B	109.3 (8)
C18—C4—C3	107.17 (6)	H17A—C17—H17B	107.7 (12)
C10—C5—C6	120.53 (6)	C15—C17—H17C	112.8 (8)
C10—C5—C4	121.86 (6)	H17A—C17—H17C	108.0 (12)
C6—C5—C4	117.47 (6)	H17B—C17—H17C	107.6 (11)
C7—C6—C5	126.80 (6)	C4—C18—H18A	111.3 (8)
C7—C6—H6A	117.7 (7)	C4—C18—H18B	109.3 (8)
C5—C6—H6A	114.9 (7)	H18A—C18—H18B	110.3 (11)
C6—C7—C8	128.23 (6)	C4—C18—H18C	108.9 (8)
C6—C7—H7A	115.8 (7)	H18A—C18—H18C	109.0 (10)
C8—C7—H7A	115.7 (7)	H18B—C18—H18C	108.0 (11)
C9—C8—C14	118.71 (6)	C4—C19—H19A	110.6 (9)
C9—C8—C7	121.08 (6)	C4—C19—H19B	109.0 (9)
C14—C8—C7	120.20 (6)	H19A—C19—H19B	109.6 (12)
C11—C9—C8	119.44 (6)	C4—C19—H19C	110.9 (8)
C11—C9—C20	122.14 (6)	H19A—C19—H19C	106.1 (12)
C8—C9—C20	118.41 (6)	H19B—C19—H19C	110.6 (12)
C5—C10—C1	121.89 (6)	C10—C20—C9	107.30 (5)
C5—C10—C20	120.26 (6)	C10—C20—H20A	108.4 (6)
C1—C10—C20	116.97 (6)	C9—C20—H20A	110.8 (6)
O2—C11—C9	122.74 (6)	C10—C20—H20B	109.8 (6)
O2—C11—C12	116.45 (5)	C9—C20—H20B	110.4 (6)
C9—C11—C12	120.63 (6)	H20A—C20—H20B	110.1 (9)
O3—C12—C11	120.14 (5)		
			
O1—C1—C2—C3	160.07 (7)	O1—C1—C10—C20	−4.42 (10)
C10—C1—C2—C3	−24.13 (10)	C2—C1—C10—C20	179.74 (6)
C1—C2—C3—C4	54.32 (9)	C8—C9—C11—O2	−175.30 (6)
C2—C3—C4—C5	−48.51 (9)	C20—C9—C11—O2	5.92 (10)
C2—C3—C4—C19	72.08 (9)	C8—C9—C11—C12	9.76 (9)
C2—C3—C4—C18	−168.92 (7)	C20—C9—C11—C12	−169.02 (6)
C19—C4—C5—C10	−108.14 (8)	O2—C11—C12—O3	−2.53 (9)
C18—C4—C5—C10	131.62 (7)	C9—C11—C12—O3	172.71 (6)
C3—C4—C5—C10	13.49 (9)	O2—C11—C12—C13	179.82 (6)
C19—C4—C5—C6	67.64 (8)	C9—C11—C12—C13	−4.93 (10)
C18—C4—C5—C6	−52.61 (8)	O3—C12—C13—C14	−178.92 (6)
C3—C4—C5—C6	−170.74 (6)	C11—C12—C13—C14	−1.26 (10)
C10—C5—C6—C7	35.04 (12)	O3—C12—C13—C15	−1.36 (10)
C4—C5—C6—C7	−140.79 (8)	C11—C12—C13—C15	176.30 (6)
C5—C6—C7—C8	−3.68 (14)	C12—C13—C14—C8	2.58 (11)
C6—C7—C8—C9	−29.82 (12)	C15—C13—C14—C8	−174.89 (7)
C6—C7—C8—C14	148.89 (8)	C9—C8—C14—C13	2.18 (10)
C14—C8—C9—C11	−8.32 (10)	C7—C8—C14—C13	−176.55 (7)
C7—C8—C9—C11	170.41 (6)	C14—C13—C15—C17	−42.19 (10)
C14—C8—C9—C20	170.51 (6)	C12—C13—C15—C17	140.37 (7)
C7—C8—C9—C20	−10.77 (9)	C14—C13—C15—C16	81.62 (9)
C6—C5—C10—C1	−159.14 (6)	C12—C13—C15—C16	−95.82 (8)
C4—C5—C10—C1	16.50 (10)	C5—C10—C20—C9	−75.85 (8)
C6—C5—C10—C20	9.77 (10)	C1—C10—C20—C9	93.60 (7)
C4—C5—C10—C20	−174.58 (6)	C11—C9—C20—C10	−106.68 (7)
O1—C1—C10—C5	164.85 (7)	C8—C9—C20—C10	74.52 (7)
C2—C1—C10—C5	−10.99 (10)		

### Hydrogen-bond geometry (Å, °)

**Table:**

Cg1 is the centroid of C8–C9/C11–C14 ring.

**Table:**

*D*—H···*A*	*D*—H	H···*A*	*D*···*A*	*D*—H···*A*
O2—H1O2···O1	0.870 (18)	2.088 (18)	2.9479 (8)	169.8 (15)
O2—H1O2···O2^i^	0.870 (18)	2.541 (16)	2.8818 (7)	104.3 (12)
O3—H1O3···O2	0.875 (14)	2.208 (16)	2.6955 (7)	114.9 (12)
O3—H1O3···O1^i^	0.875 (14)	2.046 (14)	2.8448 (7)	151.3 (14)
C7—H7A···O2^ii^	0.974 (12)	2.440 (12)	3.2262 (9)	137.5 (10)
C15—H15A···O3	1.007 (15)	2.364 (15)	2.8216 (8)	106.6 (10)
C18—H18B···O3^iii^	0.986 (15)	2.585 (15)	3.3467 (10)	134.1 (11)
C19—H19B···Cg1^ii^	1.011 (15)	2.798 (16)	3.7130 (10)	150.8 (12)
C20—H20A···Cg1^iv^	0.993 (11)	2.847 (12)	3.7506 (8)	151.6 (9)

Symmetry codes:  (i) −*x*+1/2, −*y*+1/2, −*z*+1; (ii) *x*, −*y*, *z*−1/2; (iii) −*x*+1/2, *y*−1/2, −*z*+1/2; (iv) *x*, −*y*−1, *z*−1/2.
